# Dissemination of Methicillin-Susceptible CC398 *Staphylococcus aureus* Strains in a Rural Greek Area

**DOI:** 10.1371/journal.pone.0122761

**Published:** 2015-04-02

**Authors:** Styliani Sarrou, Apostolos Liakopoulos, Markella Chasioti, Antigoni Foka, Georgios Fthenakis, Charalampos Billinis, Vassiliki Spyrou, Kleoniki Pantelidi, Angeliki Roussaki-Schulze, Vassilios Lachanas, Konstantinos Makaritsis, Charalampos Skoulakis, Georgios L. Daikos, Georgios Dalekos, Iris Spiliopoulou, Efthymia Petinaki

**Affiliations:** 1 Department of Microbiology, University Hospital of Larissa, Larissa, Greece; 2 Department of Dermatology, University Hospital of Larissa, Larissa, Greece; 3 National Staphylococcal Reference Laboratory, Department of Microbiology, School of Medicine, University of Patras, Patras, Greece; 4 Faculty of Veterinary Medicine, University of Thessaly, Karditsa, Greece; 5 Technological Education Institute of Thessaly, Larissa, Greece; 6 Department of Otorhinolaryngology, University Hospital of Larissa, Larissa, Greece; 7 First Department of Propaedeutic Medicine, University of Athens, Athens, Greece; 8 Department of Medicine and Research Laboratory of Internal Medicine, University Hospital of Larissa, Larissa, Greece; Rockefeller University, UNITED STATES

## Abstract

A large collection of *Staphylococcus aureus* including a. 745 clinically significant isolates that were consecutively recovered from human infections during 2012–2013, b. 19 methicillin-susceptible (MSSA), randomly selected between 2006–2011 from our Staphylococcal Collection, c. 16 human colonizing isolates, and d. 10 strains from colonized animals was investigated for the presence and the molecular characteristics of CC398. The study was conducted in Thessaly, a rural region in Greece. The differentiation of livestock-associated clade from the human clade was based on canSNPs combined with the presence of the φ3 bacteriophage and the *tetM*, *scn*, *sak*, and *chp* genes. Among the 745 isolates, two MRSA (0.8% of total MRSA) and thirteen MSSA (2.65% of total MSSA) were found to belong to CC398, while, between MSSA of our Staphylococcal Collection, one CC398, isolated in 2010, was detected. One human individual, without prior contact with animals, was found to be colonized by a MSSA CC398. No CC398 was identified among the 10 *S*. *aureus* isolated from animals. Based on the molecular markers, the 17 CC398 strains were equally placed in the livestock-associated and in the human clades. This is the first report for the dissemination of *S*. *aureus* CC398 among humans in Greece.

## Introduction

Since 2005, the livestock-associated clonal complex (CC) 398 *Staphylococcus aureus* have been detected mainly in pigs or other food-producing animals (cattle or poultry), as well in horses, dogs, black rats or food of animal origin [1]. These strains, that can cause mainly superficial skin and soft tissue infections (SSTIs), affect primarily individuals in direct contact with animals, e.g., farm personnel, veterinarians, veterinary students or slaughterhouse workers [1–5]. The majority of these strains are methicillin-resistant [6,7]. In 2009, a new sub-population of ST398 methicillin-sensitive *S*. *aureus* (MSSA), was observed in community households in northern Manhattan, while, soon thereafter, this clone was reported as the causal agent of necrotizing pneumonia and invasive bloodstream infections in individuals not in direct animal contact in European countries or China [8–13]. Nowadays, according to whole-genome analytical and epidemiological studies, *S*. *aureus* CC398 strains cluster into two distinct phylogenetic clades, a livestock clade and a basal human clade, that predates the livestock clade [14–16]. Main characteristics of the livestock-associated *S*. *aureus* CC398 clade is the loss of φ3 bacteriophage (ΦSa3) and the acquisition of a Tn*916*-like transposon carrying the *tet*M gene; human *S*. *aureus* CC398 clade harbours ΦSa3, a beta-haemolysin (hlb) converting phage, that carries the human-specific immune evasion cluster (IEC) of genes. IEC genes encode proteins that interact specifically with the human immune response; *chp* (chemotaxis inhibitor protein) encodes an inhibitor of chemokine responses and blocks neutrophil recruitment, *sak* (staphylokinase) encodes an inhibitor of defensins, and *scn* (staphylococcal complement inhibitor) encodes an inhibitor of complement formation and neutrophil phagocytosis [14–16]. Recently, Stegger *et al* showed that these two phylogenetic clades, the livestock-associated and the human one, can be rapidly differentiate based on single-nucleotide polymorphisms (canSNP_748, canSNP_1002 and canSNP_3737) combined with the presence of *scn* and *tetM* genes [16].

Thessaly is a rural area of Central Greece with a population of 1.200.000. Predominant activity in the region is livestock production (approx. 2.000.000 sheep, 220.000 poultry, 140.000 pigs, 132.000 cows, 3.000 horses). Objective of this study was to investigate the presence and the molecular characteristics of CC398 *S*. *aureus* (MRSA or MSSA) in a population of 745 clinical staphylococcal isolates, recovered from human infections in a rural Greek area, during 2012–2013. In addition, the existence of CC398 was also examined in a small size of nasal samples obtained from healthy individuals and from food-producing animals (mainly from sheep) the same period.

## Materials and Methods

### Ethics Statement

The research protocol concerning the human specimens was approved by the Ethics Committee of the General University Hospital of Larissa (Permit Number: 358). A written informed consent was obtained from the patients and the human individuals. The data were analyzed anonymously. In addition, samples from 512 animals (332 sheep, 100 pigs and 80 cows), randomly collected, were used in the study. The 38 farms from sheep of which samples were collected, were commercial flocks, managed semi-intensively. On the other hand, the four pig farms, included in the study, were representative of the majority of pig farms of our area, while, the median number of sows was 180 (min: 150, max: 320). In all farms, the prevailing production system catered for fattening pigs; piglets were born at those farms and weaned at the age of 3 to 4 weeks, for subsequent slaughtering at 90–120 kg live bodyweight, when 20-to 24-week-old. The flocks were under veterinary care, which was provided by private practicing veterinarians. The data of the animals were obtained from veterinarians who collected samples during standard animal care, while, the owners of the farms were asked to sign a consent form and fill in a questionnaire, before sampling. No animals were euthanised during the study and efforts taken to ameliorate animal suffering. The study did not involve any experimentation, but was based in nasal swabs, that had been collected from the sheep for routine diagnostic purposes in the participating flocks. Diagnostic veterinary procedures are not within the context of relevant EU legislation for animal experimentations (Directive 86/609/EC) and may be performed in order to diagnose animal diseases and improve animal welfare.

### Bacterial strains

The strain collection included four groups (*A*, *B*, *C*, *D*) with different origin. The main group, the group *A*, consisted of 745 *S*. *aureus* isolates (253 MRSA and 492 MSSA), consecutively collected from clinically significant samples (e.g., pus, blood) from 412 out-patients and 333 in-patients, all admitted to the five hospitals of Thessaly during the period 2012–13. In addition, in order to elucidate a. if CC398 *S*. *aureus* circulated in our area prior the study period, and b. if this clone colonizes nasal cavity of human individuals and livestock-associated animals, three other smaller groups (*B*, *C*, *D*) were also included. The group *B* consisted from 19 randomly selected MSSA from the Staphylococcal Collection of Thessaly, recovered between 2006–11 from significant specimens (abscess, blood); group *C* included 16 *S*. *aureus* (2 MRSA and 14 MSSA) isolated from nasal samples collected from 92 healthy individuals (with no staphylococcal infection during the six months previous to sampling), of which 42 people were occupational contact with animals (32 sheep farmers and 10 pig farmers); group *D* included 10 *S*. *aureus* (MSSA) recovered from 512 nasal swabs of livestock animals (332 sheep, 100 pigs and 80 cows) with no clinical staphylococcal infection. One staphylococcal isolate was tested from each person or animals. Limitation of this study is that the size of tested samples of group *B*, *C*, and *D* were small and were not estimated according to statistical rules.

During the study, demographic (name, age, gender, residence, occupation, travels, previous hospitalizations) and clinical information (underlying disease, antibiotic therapy) were collected from humans from whom samples were collected. Similar data were collected regarding animals from which samples were collected.

All nasal samples, obtained either from humans or animals, were enriched in tryptic soy broth (OXOID, Basingstoke, England) with 7% NaCl at 37°C for 24 h and then plated onto manitol salt agar (OXOID, Basingstoke, England) and cultured at 37°C for 24 h. Hospital- and community-associated strains were defined according to published criteria [17].

### Identification and susceptibility testing

Identification to species level was based on colonial morphology, Gram stain, catalase and coagulase production (Slidex Staph Plus; bioMerieux, Marcy l’Etoile, France). Susceptibility to 23 antimicrobial agents (azithromycin, benzylpenicillin, cefoxitin, clindamycin, daptomycin, erythromycin, fosfomycin, fusidic acid, gentamicin, imipenem, levofloxacin, linezolid, moxifloxacin, mupirocin, nitrofurantoin, oxacillin, rifampicin, teicoplanin, tetracycline, tigecycline, tobramycin, trimethoprim/sulfamethoxazole, vancomycin) was determined by using the VITEK-2 system (bioMerieux, Marcy l’Etoile, France), following EUCAST recommendations [18].

### Molecular methods

The initial screening test for CC398 strains was based on the presence of *aroE* allele 35, the most characteristic for the detection of this clonal complex. According to multilocus sequence typing data, this allele is present only in 1.46% of all sequence types. Complete MLST was performed to all isolates bearing *aroE* allele 35. In all CC398 strains, *agr* groups, SCC*mec* types, *spa* types and genes encoding PVL (*lukS/lukF*-PVL) and toxic shock syndrome toxin-1 (*tst*) were also defined by using PCRs [19]. Moreover, antimicrobial resistance genes (*mecA*, *mecC*, *ermA*, *ermB*, *ermC*, *ermT*, *lnuA*, *lnuB*, *lnuC*, *lnuD*, *vgaA*, *tetM*, *tetL*, *tetO*, *tetK*, *dfrD*, *dfrK*, *dfrG*, and *dfrS1)* were detected by PCR, as described before [19, 20]. The differentiation between livestock-associated and human CC398 strains was based a. on the determination of canSNP_748, canSNP_1002 and canSNP_3737 as described by Stegger *et al*, b. on the presence of ΦSa3 confirmed by *integrase3* gene (φ3*int*) PCR amplification combined with the absence of functional beta-hemolysin gene (*hlb*), and c. on the presence of *scn*, *chp* and *sak* genes by PCR [16,21,22]. To obtain information on the location of IEC genes, PCRs were performed using relevant combinations of specific primers for *scn*, *sak* and *chp*, as previously reported by van Wamel *et al* [22]. PCR products were sequenced and results obtained were compared with the *Staphylococcus aureus* phage phiNM3 complete genome (DQ530361).

## Results

According to screening, *aro35* allele was detected in 17 isolates: 15 isolates of group A (15/745), one of group B (1/19) and one of group C (1/16). No isolate was found to be *aro35-* positive from group D (0/10).

The complete MLST scheme revealed that all these 17 isolates belonged to CC398. The majority of them (13 isolates) was ST398, whereas, four strains belonged to new variants, namely ST2684 (two isolates), ST2759 (one isolate) or ST2978 (one isolate) (Table 1). Eleven of these isolates were recovered from SSTIs or abscesses, five from bloodstream infections and one from nares (Table 1). All CC398 strains belonged to *agr*1 and distributed in seven *spa* types (Table 1). According to patients’ demographic data, only four strains could be classified as hospital-associated (Table 1). All people were adults; none had reported prior contact and occupation with animals or travel in countries with an increased prevalence of ST398.

**Table 1:** Characteristics of *Staphylococcus aureus* CC398 strains isolated in Central Greece.

Strain	Origin (CA/HA)	Isolation date	Ward	ST/agr	spa/SCC*mec*	Antimicrobial resistance phenotype*	Resistance genes detected	*canSNPs* 748,1002,3737	φ*3int/hlb*	*chp/sak/ scn/*	*clade*
1685-MSSA	SSTI/CA	01-03-2010	Internal medicine	2684/I	t034,	P, E, CC, TET, SXT	*ermC*, *tetM*, *dfrG*	TCA,ATA,GAG	-,+	-,-,-	animal
2682- MSSA	pus/CA	27-03-2012	Vascular surgery	398/I	t034	P,CC, TET,SXT	*lnuB*, *tetM*, *dfrG*	TCA,ATA,GAG	-,+	-,-,-	animal
3125-MSSA	SSTI/CA	09-10-2012	Orthopaedics	398/I	t034	P,CC, TET	*lnuB*, *tetM*,	TCA,ATA,GAG	-,+	-,-,-	animal
3130-MSSA	abscess/HA	13-10-2012	Otorhinolaryngology	398/I	t034	P, FOS, TET	*tetM*	TCA,ATA,GAG	-,+	-,-,-	animal
3221-MRSA	SSTI/CA	16-10-2012	Dermatology	398/I	t034/V	P, OX, E, CC, RA, TET, SXT	*mecA*, *ermA*, *tetM*, *dfG*	TCA,ATA,GAG	-,+	-,-,-	animal
3158-MSSA	abscess/CA	24-10-2012	Ophthalmology	2684/I	t011	P, SXT	*dfrG*	TCA,ATA,GAG	-,+	-,-,-	animal
3303-MRSA	abscess/CA	20-12-2012	Internal medicine	398/I	t011/V	P, OX, CC, TET	*mecA*, *lnuA*, *vgaA*, *tetM*	TCA,ATA,GAG	-,+	-,-,-	animal
3343-MSSA	SSTI/CA	12-01-2013	Orthopaedics	398/I	t1255	-	-	CCA,CTA,GGG	+,-	+,-,+	human
3402-MSSA	SSTI/CA	01-02-2013	Internal medicine	398/I	t1451	E,CC,	*ermT*, *ermC*,	CCA,CTA,GGG	+,-	+,-,+	human
3659-MSSA	abscess/CA	11-05-2013	Otorhinolaryngology	2759/I	t6606	E, CC	*ermT*	CCA,CTA,GGG	+,-	+,+, -	human
3699-MSSA	blood/HA	22-05-2013	Internal medicine	398/I	t468	E, CC	*ermT*,	CCA,CTA,GGG	+,-	+,-,+	human
4013-MSSA	blood/CA	29-09-2013	Dermatology	398/I	t1451	-	-	CCA,CTA,GGG	+,-	+,-,+	human
4042-MSSA	blood/HA	09-10-2013	Internal medicine	2759/I	t6606	E, CC	*ermT*	CCA,CTA,GGG	+,-	+,+,-	human
4066-MSSA	blood/CA	19-10-2013	Internal medicine	398/I	t6606	E,CC	*ermT*,	CCA,CTA,GGG	+,-	+,+,-	human
4218-MSSA	blood/HA	13-12-2013	Internal medicine	398/I	t011	P, TET	*tetM*,	TCA,ATA,GAG	-,+	-,-,-	animal
4219-MSSA	SSTI/CA	13-12-2013	Dermatology	398/I	t034	P, FOS, TET	*tetM*	TCA,ATA,GAG	-,+	-,-,-	animal
29-MSSA	nasal carriage	15-10-2013	Out-patient	398/I	t13982	E,CC	*ermT*,	CCA,CTA,GGG	+,-	+,-,+	human

CA: community–associated; HA: hospital-associated; ST: sequence type; SSTI: skin and soft tissue infection; P: penicillin; FOS: fosfomycin; E: erythromycin; CC: clindamycin; SXT: sulfamethoxazole-trimethoprim; RA: rifampicin; OX: oxacillin.

*All isolates were susceptible to vancomycin, teicoplanin, fusidic acid, linezolid, daptomycin, tobramycin, gentamicin, levofloxacin, moxifloxacin.

Susceptibility testing results are in Table 1. Two strains were oxacillin-resistant and carried *mecA* gene; resistance to erythromycin was associated with presence of *ermA*, *ermC* and *ermT*, resistance to lincomycin with presence of *lunA*, *lnuB* and *vgaA*, resistance to tetracycline with presence of *tetM* gene and resistance to sulfamethoxazole-trimethoprim with presence of *dfrG*. No strain carried either *lukS/lukF*-PVL or *tst* genes.

According to canSNP assays, nine strains were placed in the livestock clade (see Table 1). Eight of them were tetracycline-resistant and carried the *tetM* gene, while, only two were MRSA. As anticipated, all were negative for the presence of ΦSa3 and belonged to *spa* t011 or t034, types strongly related to the livestock-associated clade as previously reported [16]. On the other hand, according to canSNPs, eight strains were placed in the human clade; their *spa* types were t468, t1255, t1451, t13982 and t6606 (see Table 1). All of them were tetracycline susceptible and carried the ΦSa3 with various combinations of the *scn*, *chp* and *sak* genes; five strains were *scn+/ chp+*, while the remaining three strains were *sak+/ chp+*(see Table 1). This latter combination is described for the first time, while both genes were located to ΦSa3 [22, 23]. All erythromycin- resistant strains of the human clade carried the *ermT* gene.

## Discussion

Although the molecular epidemiology of MRSA in Greece has been investigated, there was a limited surveillance of MSSA [17, 24]. Until now, one MRSA ST398 has been detected in 2009 in Athens, as well as one MSSA, details of which have been reported before. Both isolates were recovered from blood samples [13]. To the best of our knowledge, this is the first report from Greece indicating that, during 2012–2013, among a large collection of 745 clinical staphylococcal isolates from infected humans, 0.8% of MRSA (2/253 isolates in group A) and 2.65% of MSSA (13/492 isolates in group A) belonged to CC398. In addition, one human individual, without prior contact with animals, was found to be colonized by a CC398 MSSA.

Based on the canSNPs combined with the presence of ΦSa3 nine and eight isolates were placed in the livestock and in the human clade, respectively. Our results were in concordance with previous reports, that have demonstrated that, the detection of some resistant genes, such as the *tetM* and the *ermT* genes, could be used as rapid tools for differentiate livestock-associated clade from the human CC398 clade [25, 26].

It was surprising to find that in samples collected from animals (group D) no CC398 *S*. *aureus* was found. This could be explained by firstly the majority of the animals tested were sheeps and secondary the size of tested samples was very small. So, no concrete conclusions about the existence of CC398 in animal populations (D) could be obtained. However, the detection of one livestock-associated CC398 MSSA, *spa* t034 in 2010, indicates that the livestock clade already existed in the area and indicates the need for a wider surveillance in collaboration with veterinary researchers, which is in progress.

In conclusion, the present study underscores the presence of the two major host-associated *S*. *aureus* CC398 clades in a rural Greek area. The majority of isolates was MSSA and caused a large spectrum of human infections. Although, the prevalence rate of CC398 MSSA was not found to be high (2.65%) as compared to those reported from other countries [25, 26], the presence of this clone in patients and healthy individuals emphasizes the need of epidemiological surveillance for MSSA.
